# Policy Design and Consumer Direction: Cross-country Comparisons on Consumer-directed Care Programs

**DOI:** 10.1093/geroni/igab046.3033

**Published:** 2021-12-17

**Authors:** Jinbao ZHANG, Julia Shu-Huah WANG, Yu-Chih Chen

**Affiliations:** 1 University of Hong Kong, Hong Kong, Hong Kong; 2 The University of Hong Kong, Hong Kong, Hong Kong; 3 University of Hong Kong, Pok Fu Lam, Hong Kong

## Abstract

Objectives: The consumer-directed care (CDC) program aims to maximize health outcomes by offering older adults more control, choice, and flexibility over the care services they received. However, countries may operate CDC programs in different ways based on heterogenous sociostructural systems. We proposed a comparative framework to evaluate three dimensions of CDC—control and direct services, variety of service options, and information and support—and analyzed how countries varied in their policy design to achieve consumer direction. Methods: Using cross-national document analysis, we analyzed eleven CDC programs from seven selected countries (Netherlands, United States (US), United Kingdom (UK), Germany, China, Australia, and Spain) with five CDC care regimes. A total of fourteen indicators capturing three dimensions of CDC programs was developed. We further used these indicators to evaluate and compare similarities and differences of policy features across countries using descriptive statistics and graphical approaches. Results: CDC programs in the Netherlands, Arkansas, and the UK ranked at the top in consumer direction. All countries except Germany employed a “service-based” principle in determination of service type. Training care workers was in the most widespread use to assure quality of care. Merely the UK and Germany integrated CDC and conventional agency care without restrictions. Representative with relevant support was only available in the UK and Netherlands. Discussion and Implication: CDC models involve multi-faced aspects, rather than dichotomies and discrete entities. Implications include the need for a systematic reflection with our developed framework and enriching variety of service options to promote consumer direction.

